# Neuroimaging Correlates of Cognitive Deficits in Wilson's Disease

**DOI:** 10.1002/mds.29123

**Published:** 2022-06-20

**Authors:** Samuel Shribman, Maggie Burrows, Rhian Convery, Martina Bocchetta, Carole H. Sudre, Julio Acosta‐Cabronero, David L. Thomas, Godfrey T. Gillett, Emmanuel A. Tsochatzis, Oliver Bandmann, Jonathan D. Rohrer, Thomas T. Warner

**Affiliations:** ^1^ Reta Lila Weston Institute UCL Queen Square Institute of Neurology London; ^2^ Dementia Research Centre UCL Queen Square Institute of Neurology London United Kingdom; ^3^ MRC Unit for Lifelong Health and Ageing University College London London United Kingdom; ^4^ Centre for Medical Image Computing University College London London United Kingdom; ^5^ Biomedical Engineering & Imaging Sciences King's College London London United Kingdom; ^6^ Tenoke Ltd Cambridge United Kingdom; ^7^ Neuroradiological Academic Unit UCL Queen Square Institute of Neurology London United Kingdom; ^8^ Wellcome Centre for Human Neuroimaging UCL Queen Square Institute of Neurology London United Kingdom; ^9^ Department of Clinical Chemistry Northern General Hospital Sheffield United Kingdom; ^10^ UCL Institute of Liver and Digestive Health and Royal Free Hospital London United Kingdom; ^11^ Sheffield Institute of Translational Neuroscience Sheffield United Kingdom

**Keywords:** Wilson's disease, cognition, magnetic resonance imaging

## Abstract

**Background:**

Cognitive impairment is common in neurological presentations of Wilson's disease (WD). Various domains can be affected, and subclinical deficits have been reported in patients with hepatic presentations. Associations with imaging abnormalities have not been systematically tested.

**Objective:**

The aim was to determine the neuroanatomical basis for cognitive deficits in WD.

**Methods:**

We performed a 16‐item neuropsychological test battery and magnetic resonance brain imaging in 40 patients with WD. The scores for each test were compared between patients with neurological and hepatic presentations and with normative data. Associations with Unified Wilson's Disease Rating Scale neurological examination subscores were examined. Quantitative, whole‐brain, multimodal imaging analyses were used to identify associations with neuroimaging abnormalities in chronically treated stable patients.

**Results:**

Abstract reasoning, executive function, processing speed, calculation, and visuospatial function scores were lower in patients with neurological presentations than in those with hepatic presentations and correlated with neurological examination subscores. Deficits in abstract reasoning and phonemic fluency were associated with lower putamen volumes even after controlling for neurological severity. About half of patients with hepatic presentations had poor performance in memory for faces, cognitive flexibility, or associative learning relative to normative data. These deficits were associated with widespread cortical atrophy and/or white matter diffusion abnormalities.

**Conclusions:**

Subtle cognitive deficits in patients with seemingly hepatic presentations represent a distinct neurological phenotype associated with diffuse cortical and white matter pathology. This may precede the classical neurological phenotype characterized by movement disorders and executive dysfunction and be associated with basal ganglia damage. A binary phenotypic classification for WD may no longer be appropriate. © 2022 The Authors. *Movement Disorders* published by Wiley Periodicals LLC on behalf of International Parkinson and Movement Disorder Society

Wilson's disease (WD) is an autosomal‐recessive disorder of copper metabolism that presents with movement disorders, psychiatric features, or liver disease.^1^ Cognitive impairment is also common among patients with neurological presentations but usually mild.^2^, ^3^, ^4^ Various domains, including abstract reasoning, executive function, memory, processing speed, visuospatial function, and social cognition, can be affected.^2^, ^3^, ^4^, ^5^, ^6^, ^7^, ^8^, ^9^, ^10^, ^11^, ^12^ Subclinical deficits have also been reported in patients with hepatic presentations without encephalopathy.^12^, ^13^, ^14^ Understanding the anatomical basis for cognitive symptoms may provide insights into the evolution of neurological involvement in WD.

Associations between cognitive impairment and neuroimaging abnormalities in WD have been examined only in a few studies. Seniów *et al* found that patients with T2‐weighted signal abnormalities localized to the basal ganglia had similar cognitive abilities to those with more diffuse abnormalities.^3^ They concluded that basal ganglia dysfunction is the primary cause of cognitive impairment in WD. Frota *et al* demonstrated that patients with deficits in multiple domains had higher scores on a semiquantitative magnetic resonance imaging (MRI) scale.^2^ Using quantitative analyses, Dong *et al* reported that measures of prospective memory correlate with diffusion abnormalities in specific white matter tracts and cortical thickness in the right orbitofrontal gyrus.^15^, ^16^ The extent to which pathology within and beyond the basal ganglia contributes to other aspects of cognitive function in WD remains unclear.

We used a combination of quantitative, whole‐brain analyses on T1‐weighted fluid‐attenuated inversion recovery (FLAIR), diffusion‐weighted imaging (DWI), and susceptibility‐weighted imaging (SWI) sequences to identify imaging correlates of neurological involvement and copper indices in 40 prospectively recruited patients with WD.^17^ Here, we aimed to apply these methods in combination with a comprehensive battery of neuropsychological tests to determine the anatomical basis for cognitive deficits in the same cohort.

## Patients and Methods

### Study Population

Forty patients were recruited to a prospective study on WD.^17^, ^18^ Consecutive patients attending neurology, hepatology, and metabolic clinics at the National Hospital for Neurology and Neurosurgery (NHNN) and Royal Free Hospital and members of the Wilson's Disease Support Group UK research register were invited to participate. We included patients aged 16 years or above who satisfied the Leipzig diagnostic criteria.^19^ All participants provided written informed consent. The study was approved by the regional ethics committee (18/NE/0279).

### Clinical Assessments

Participants attended research visits at NHNN for clinical assessments and neuroimaging between January and December 2019. Participants were divided into neurological and hepatic presentations according to the international consensus on the phenotypic classification of WD.^19^ They were subcategorized according to recent neurological status. Those with neurological presentations or deterioration related to nonadherence to chelation therapy in the preceding 6 months were classified as having active, as opposed to stable, neurological disease. Unified Wilson's Disease Rating Scale neurological examination (UWDRS‐N) subscores were recorded as a measure of movement disorder severity.^20^

A 16‐item neuropsychological test battery designed to test a range of cognitive domains was applied. Abstract reasoning was tested using the Weschler Abbreviated Scale of Intelligence Matrix Reasoning Test (MRT), and language was tested using the National Adult Reading Test and Graded Naming Test. Memory was tested using the Recognition Memory Test for Faces (RMTF), Recognition Memory Test for Words (RMTW), and Paired Associate Learning Test (PALT). Processing speed was tested using the Trail Making Test Part A (TMTA), and executive function was tested using the Weschler Memory Scale Revised Digit Span Backwards (DSB), phonemic fluency test (FAS), semantic fluency test (animals), Delis–Kaplan Executive Function System Color‐Word Interference Test, Trail Making Test Part B (TMTB), and Weschler Adult Intelligence Scale Digit Symbol (DSym) test. Calculation was tested using the Graded Difficulty Arithmetic (GDA) test, and visuoperceptual and visuospatial abilities were tested using the Visual Object and Space Perception Battery Fragmented Letters and Visual Object and Space Perception Battery Number Location (VOSPNL) tests, respectively. Social cognition was tested using the Ekman Facial Emotion Recognition (Ekman) test. Testing took between 60 and 90 minutes. All participants took at least one break. Raw scores were converted to *z* scores using normative data from the sources outlined in Table S1.

### Imaging Acquisition, Processing, and Analysis

T1‐weighted (structural), FLAIR, DWI, and SWI data were acquired on a Siemens Prisma 3T system with a 64‐channel head/neck coil using the pulse sequence parameters in Table S2. Data were visually inspected after each acquisition to allow individual sequences to be repeated if movement artefacts were identified. Associations with neuropsychological test scores were tested in stable patients for each sequence using methods described in a previous publication.^17^ Age and sex were used as covariates in all analyses. Where associations were identified, the analyses were repeated, including UWDRS‐N as a covariate. Additional details on the following methods described are provided in Appendix S1.

### T1‐Weighted Imaging

Voxel‐based morphometry (VBM) was performed using Statistical Parametric Mapping (SPM12, version 7771, http://www.fil.ion.ucl.ac.uk/spm).^21^ T1‐weighted images were segmented and spatiallynormalized.^22^ All segmentations were visually checked for quality. Grey Matter (GM) and White Matter (WM) segments were transformed, modulated, and smoothed to create preprocessed GM tissue maps. These were fitted to multiple regression analyses to test associations with neuropsychological test scores. Total intracranial volume (TIV) was included as a covariate in addition to age and sex.^23^ Statistical thresholds were set at *P* < 0.05 for family‐wise error (FWE) correction.

Region‐of‐interest (ROI) analyses were also performed given subcortical regions are susceptible to systematic misregistration errors when spatially normalizing structurally abnormal brains. T1‐weighted images were parcellated using the Geodesic Information Flow pipeline.^24^ The brainstem was segmented using FreeSurfer.^25^ The volume of eight subcortical ROI was extracted and expressed as a percentage of TIV. Linear regression was performed in R (version 3.6.0, http://www.R-project.org). *P*‐values with and without false discovery rate (FDR) correction were calculated.

### Diffusion‐Weighted Imaging

Functional MRI of the Brain Software Library (version 6.0.3, https://fsl.fmrib.ox.ac.uk/fsl) was used to preprocess DWI data before tensor fitting to generate diffusion tensor imaging (DTI) data. Tract‐based spatial statistics was used to test associations between fractional anisotropy (FA), mean diffusivity (MD), axial diffusivity (AD), and radial diffusivity (RD) in WM tracts and neuropsychological test scores. Design matrices were generated using the general linear model, and RANDOMISE was used to perform nonparametric permutation analyses.^26^ Threshold‐free cluster enhancement was used with FWE‐corrected *P* < 0.05.^27^

### FLAIR Imaging

Hyperintense signal abnormalities were segmented using Bayesian model selection, an automated lesion segmentation tool applied to rigidly coregistered T1‐weighted and FLAIR sequences.^28^ The volume of these abnormalities within 40 anatomically defined regions was calculated and log_e_‐transformed to reduce skewness.^29^ A linear regression model was used to test the associations between the log_e_‐transformed volumes in each region and neuropsychological test scores in R. TIV was included as a covariate of no interest in addition to age and sex. *P*‐values for coefficients of interest were calculated using FDR correction.

### Susceptibility‐Weighted Imaging

Quantitative susceptibility maps (QSM) were reconstructed from susceptibility‐weighted images using a Multi‐Scale Dipole Inversion–based pipeline in QSMbox (https://gitlab.com/acostaj/QSMbox).^30^ Whole‐brain analyses were then performed with the QSMexplorer pipeline (https://gitlab.com/acostaj/QSMexplorer).^31^ Absolute susceptibility maps were used to identify the associations with neuropsychological test scores. RANDOMISE was used to perform nonparametric permutation analyses. Threshold‐free cluster enhancement was enabled to identify clusters of voxels with FWE‐corrected *P* < 0.05.

### Statistical Analysis

Group differences in demographic and clinical characteristics were tested in R. *z* Scores for each neuropsychological test were compared between patients with neurological and hepatic presentations using the Mann‐Whitney *U* test. Spearman's correlation coefficients were used to measure the associations between individual tests and UWDRS‐N scores in stable patients. *P‐*values with and without FDR correction were recorded.

### Data Sharing

Anonymized data are available on request to the corresponding author.

## Results

### Demographic and Clinical Characteristics

The cohort consisted of 23 patients with neurological and 17 patients with hepatic presentations. Five patients were classified as having active disease. The mean age was 43 years (range: 16–68), and disease duration was 23 years. UWDRS‐N scores were higher in patients with neurological presentations (22, interquartile range [IQR]: 14–37) than hepatic presentations (3, IQR: 0–4; *P* < 0.001) and patients with active disease (48, IQR: 40–51) than stable disease (9, IQR: 3–17; *P* = 0.001), as expected. The most frequent examination findings in patients with neurological presentations were impaired finger taps (87%), leg agility (83%), arm and hand dystonia (83%), oromandibular dystonia (78%), rapid alternating movements (74%), handwriting (74%), and speech (70%). There were no differences in age, sex, disease duration, cirrhosis, or treatments between patients with neurological and hepatic presentations. Further details on demographic and clinical characteristics are provided in Table S3.

### Neuropsychological Testing

Neuropsychological testing was performed in 39 participants. One declined, and several were unable to complete specific tests due to severe dysarthria, impaired upper‐limb function, or language barrier such that 32 of 780 (4%) test scores were missing or excluded.

Group differences in neuropsychological test scores and associations with UWDRS‐N scores are summarized in Table 1. The neurological group performed worse than the hepatic group in the MRT, TMTA, and VOSPNL after FDR correction. Scores for other tests of executive function, including DSB, FAS, TMTB, and DSym, were also lower in the neurological group. These differences did not persist after controlling for multiple comparisons. UWDRS‐N scores were negatively correlated with MRT, RMTF, TMTA, DSym, and GDA scores after FDR correction. There were no differences in test scores between patients with and without cirrhosis (*P* < 0.05).

**TABLE 1:** Group differences and associations with UWDRS‐N for neuropsychological test scores

Domain	Test	Hepatic (n = 17) *z* score, median [IQR]	Neurological (n = 22) *z* score, median [IQR]	*P‐*value	UWDRS‐N (n = 35) Spearman's coefficient	*P‐*value
Abstract reasoning	MRT	1.2 [1.0, 1.6]	0.1 [−1.0, 1.2]	0.001**	−0.49	0.001**
Language	NART	0.8 [0.3, 1.5]	0.7 [−0.8, 1.0]	0.33	−0.14	0.44
	GNT	0.4 [−0.9, 0.9]	−0.4 [−1.9, 0.6]	0.31	−0.08	0.65
Memory	RMTF	−0.8 [−1.9, 0.4]	−1.6 [−3.0, −0.1]	0.11	−0.47	0.002**
	RMTW	0.9 [0.6, 1.0]	0.9 [0.5, 1.2]	0.66	−0.20	0.23
	PALT	−0.4 [−0.9, 0.1]	−0.2 [−2.0, 0.7]	0.75	0.10	0.56
Processing speed	TMTA	−0.2 [−0.9, 0.8]	−1.6 [−2.7, −0.8]	0.002**	−0.52	0.001**
Executive function	DSB	0.9 [−0.8, 1.8]	−0.8 [−0.8, 0.1]	0.05*	−0.21	0.21
	FAS	0.3 [−0.7, 1.1]	−1.0 [−1.5, 0.1]	0.02*	−0.38	0.03*
	Animals	0.6 [−0.9, 1.5]	0.2 [−0.8, 1.2]	0.67	−0.32	0.08
	DKEFSI	0.3 [0.0, 1.0]	−0.3 [−1.3, 1.0]	0.21	−0.31	0.06
	TMTB	0.3 [−1.0, 0.7]	−1.4 [−6.2, −0.1]	0.03*	−0.34	0.04*
	DSym	0.7 [−0.3, 1.0]	−0.3 [−1.3, 0.0]	0.01*	−0.46	0.004**
Calculation	GDA	0.7 [0.0, 1.3]	0.0 [−0.9, 0.3]	0.02*	−0.44	0.008**
Visuoperceptual	VOSPFL	0.9 [0.9, 0.9]	0.9 [−0.9, 0.9]	0.86	0.05	0.76
Visuospatial	VOSPNL	0.5 [0.5, 0.5]	−0.4 [−0.4, 0.5]	0.006**	−0.37	0.02*
Social	Ekman	0.3 [−0.4, 0.9]	−0.6 [−1.5, 0.5]	0.11	−0.29	0.07

Group differences in *z* scores and associations with UWDRS‐N subscores in stable patients for each neuropsychological test are shown.

*P‐value <0.05; **P‐value <0.01.

*P‐*values less than 0.05 after FDR correction are in bold font.

UWDRS‐N, Unified Wilson's Disease Rating Scale neurological examination subscore; IQR, interquartile range; MRT, Weschler Abbreviated Scale of Intelligence Matrix Reasoning Test; NART, National Adult Reasoning Test; GNT, Graded Naming Test; RMTF, Recognition Memory Test for Faces; RMTW, Recognition Memory Test for Words; PALT, Paired Associate Learning Test; TMTA, Trail Making Test Part A; DSB, Weschler Memory Scale Revised Digit Span Backwards; FAS, phonemic fluency test; Animals, semantic fluency test; DKEFSI, Delis–Kaplan Execution Function System Color‐Word Interference subtest; TMTB, Trail Making Test Part B; DSym, Weschler Adult Intelligence Scale Digit Symbol test; GDA, Graded Difficulty Arithmetic test; VOSPFL, Visual Object and Space Perception Battery Fragmented Letters test; VOSPNL, Visual Object and Space Perception Battery Number Location test; Ekman, Ekman Facial Emotion Recognition test; FDR, false discovery rate.

**FIG 1:**
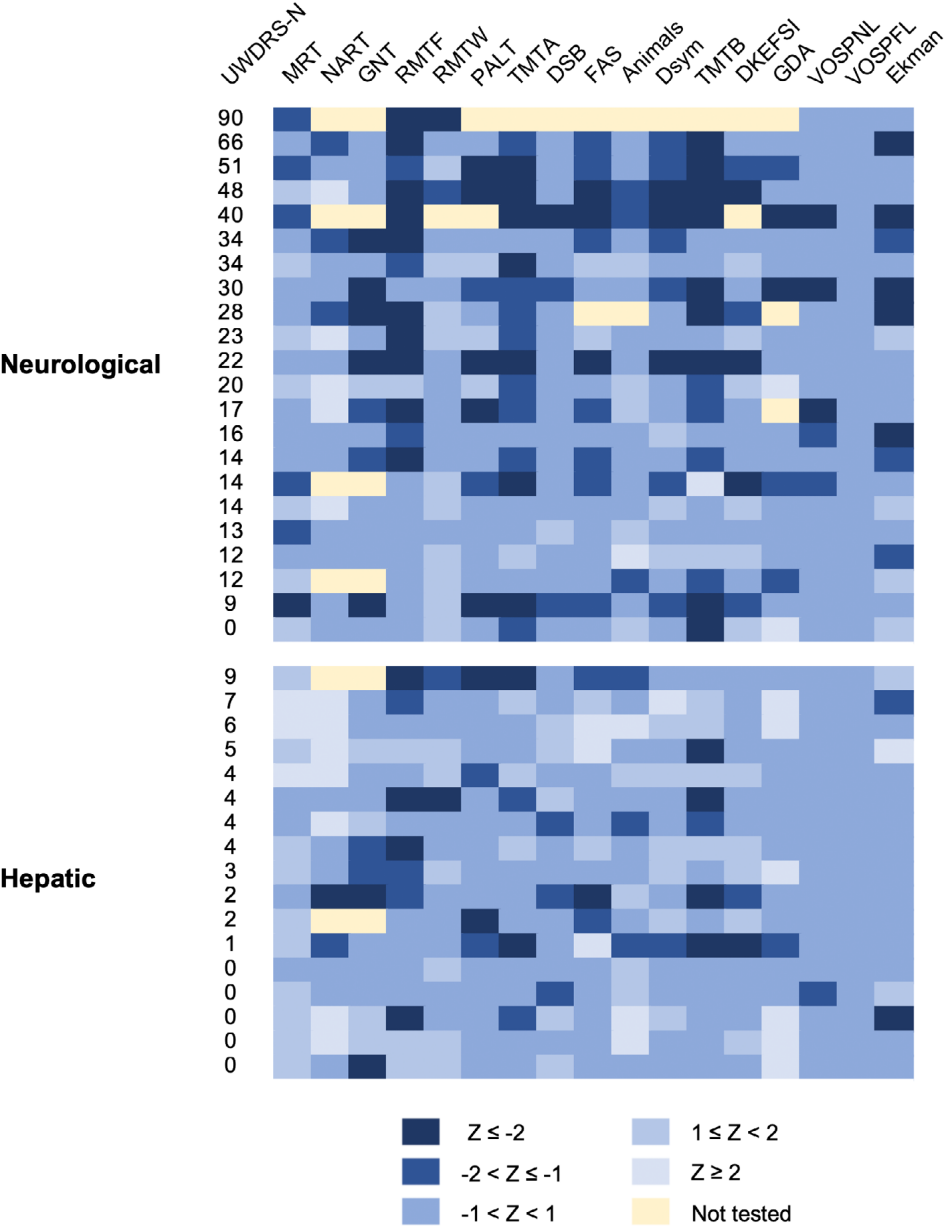
Individual participant scores for each neuropsychological test. Participants are divided into neurological and hepatic presentations and ordered according to UWDRS‐N (Unified Wilson's Disease Rating Scale neurological examination) subscores within these groups. *z* Scores for neuropsychological tests are color coded with darker shades of blue, indicating poorer performance. [Color figure can be viewed at wileyonlinelibrary.com]

Individual neuropsychological test scores are shown in Figure 1, and the frequencies of participants who scored more than two standard deviations (SDs) below the mean for each test are presented in Table S4. Low performance was relatively common for the RMTF, PALT, and TMTB irrespective of presentation. For the RMTF, 24% of patients with hepatic presentations and 45% of patients with neurological presentations scored more than two SDs below the mean. Overall, 77% of patients with neurological presentations and 53% of patients with hepatic presentations scored below this cutoff on at least one neuropsychological test. Of the five patients with hepatic presentations and UWDRS‐N scores of zero, two had scores below this cutoff in at least one neuropsychological test.

### Neuroimaging

One participant declined to undergo MRI, and another had only T1‐weighted acquisitions. Two more patients did not complete the DWI acquisitions.

Associations between neuropsychological test scores and ROI volumes are presented in Table 2. Scores for the MRT, FAS, and TMTB correlated with caudate, putamen, and pallidum volumes, respectively, after FDR correction. Associations between scores for the MRT and FAS and putamen volume persisted after UWDRS‐N scores were included as a covariate, as presented in Table S5.

**TABLE 2:** Associations between ROI volumes and neuropsychological test scores

Domain	Test	Caudate	Putamen	Pallidum	Thalamus	Amygdala	Midbrain	Pons	Cerebellum
Abstract reasoning	MRT	0.006**	<0.001***	0.001**	0.03*	0.30	0.09	0.10	0.004**
Language	NART	0.06	0.04*	0.03*	0.25	0.81	0.19	0.39	0.09
	GNT	0.03*	0.04*	0.01*	0.03*	0.96	0.16	0.20	0.05
Memory	RMTF	0.03*	0.02*	0.05*	0.31	0.44	0.08	0.10	0.07
	RMTW	0.16	0.34	0.37	0.64	0.43	0.51	0.39	0.59
	PALT	0.11	0.30	0.50	0.92	0.52	1.00	0.64	0.74
Processing speed	TMTA	0.04*	0.08	0.10	0.29	0.92	0.29	0.25	0.39
Executive function	DSB	0.06	0.02*	0.02*	0.25	0.69	0.56	0.45	0.28
	FAS	0.009**	<0.001***	0.006**	0.06	0.32	0.01*	0.01*	0.02*
	Animals	0.12	0.21	0.18	0.78	0.68	0.98	0.48	0.03*
	DKEFSI	0.16	0.07	0.13	0.41	0.38	0.37	0.46	0.16
	TMTB	0.003**	0.003**	0.001*	0.004**	0.81	0.001**	0.004**	0.35
	DSym	0.02*	0.02	0.02*	0.07	0.51	0.04*	0.06	0.05
Calculation	GDA	0.09	0.10	0.10	0.81	0.97	0.81	0.71	0.24
Visuoperceptual	VOSPFL	0.36	0.72	0.79	0.33	0.33	0.45	0.54	0.26
Visuospatial	VOSPNL	0.39	0.35	0.52	0.66	0.80	0.86	0.92	0.21
Social	Ekman	0.56	0.12	0.32	0.49	0.25	0.09	0.30	0.21

*P‐*values for coefficients when testing associations between neuropsychological test scores and ROI volumes using linear regression are shown.

Corresponding coefficients where *P* < 0.05 were positive.

*P‐value <0.05; **P‐value <0.01; ***P‐value <0.001.

*P‐*values less than 0.05 after FDR correction are in bold font.

ROI, region of interest; MRT, Weschler Abbreviated Scale of Intelligence Matrix Reasoning Test; NART, National Adult Reasoning Test; GNT, Graded Naming Test; RMTF, Recognition Memory Test for Faces; RMTW, Recognition Memory Test for Words; PALT, Paired Associate Learning Test; TMTA, Trail Making Test Part A; DSB, Weschler Memory Scale Revised Digit Span Backwards; FAS, phonemic fluency test; Animals, semantic fluency test; DKEFSI, Delis–Kaplan Execution Function System Color‐Word Interference subtest; TMTB, Trail Making Test Part B; DSym, Weschler Adult Intelligence Scale Digit Symbol test; GDA, Graded Difficulty Arithmetic test; VOSPFL, Visual Object and Space Perception Battery Fragmented Letters test; VOSPNL, Visual Object and Space Perception Battery Number Location test; Ekman, Ekman Facial Emotion Recognition test; FDR, false discovery rate.

**FIG 2:**
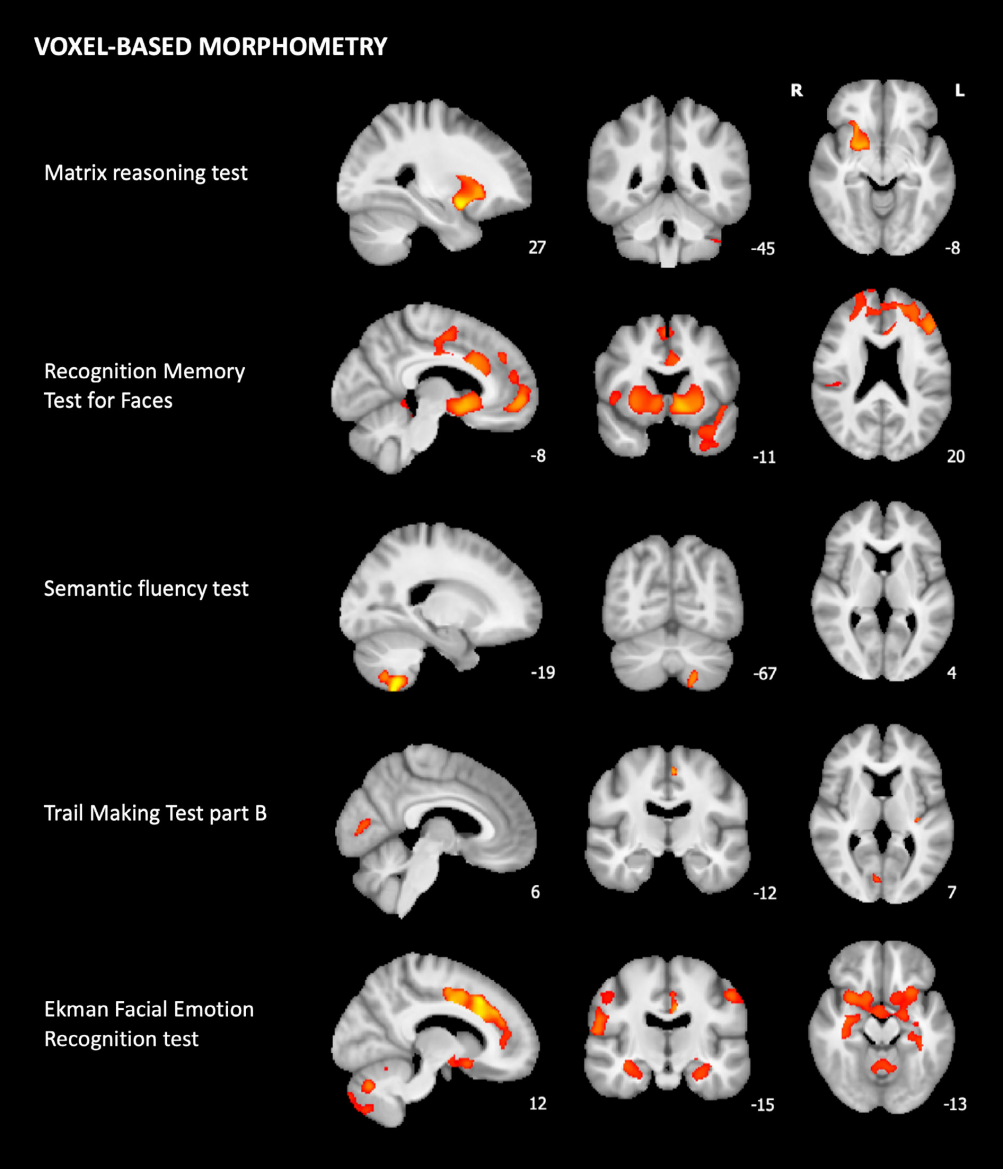
Voxel‐based morphometry for associations with neuropsychological test scores. Tissue maps show clusters where gray matter volumes decrease with worsening cognitive performance for FWE (family‐wise error)‐corrected *P*‐values <0.05. Clusters are overlaid onto the study‐wise mean template. For visualization, one slice in each of the sagittal (*x*), coronal (*y*), and axial (*z*) planes was selected, and Montreal Neurological Institute (MNI) coordinates are provided. [Color figure can be viewed at wileyonlinelibrary.com]

VBM analyses identified the associations between several neuropsychological test scores and distinct patterns of reduced GM volume, as shown in Figure 2. Clusters that persisted after including UWDRS‐N as a covariate are shown in Figure S1, and cluster‐specific statistics are provided in Table S6. MRT scores were associated with reduced GM volumes in the right putamen, insula, and orbitofrontal cortices, and animal scores were associated with reduced GM volumes in the left cerebellum. RMTF scores were associated with decreased GM volumes in diffuse, predominantly anterior cortical regions, including the bilateral cingulate, paracingulate and insula cortices, middle frontal gyri and supplementary motor area, and right superior and middle temporal gyri and subcallosal and opercular cortices. Subcortical clusters, including the caudate, putamen, dorsal midbrain, and right cerebellum, were also identified. Ekman scores were also associated with decreased GM volumes in anterior and subcortical cortical regions. These included the bilateral cingulate, paracingulate, insula and orbitofrontal cortices and pre‐central and post‐central gyri, right central opercular cortex, frontal pole and middle frontal gyrus and left superior frontal gyrus, and temporal fusiform cortex. Subcortical clusters in the bilateral hippocampus and cerebellum and right putamen were identified. Clusters in the bilateral cingulate and paracingulate cortices and right central opercular cortex persisted after including UWDRS‐N score as a covariate. TMTB scores were associated with decreased volume in the left insula cortex, supplementary motor area, precuneus and occipital fusiform gyrus, and right intracalcarine cortex. These clusters also persisted when UWDRS‐N was included as a covariate.

**FIG 3:**
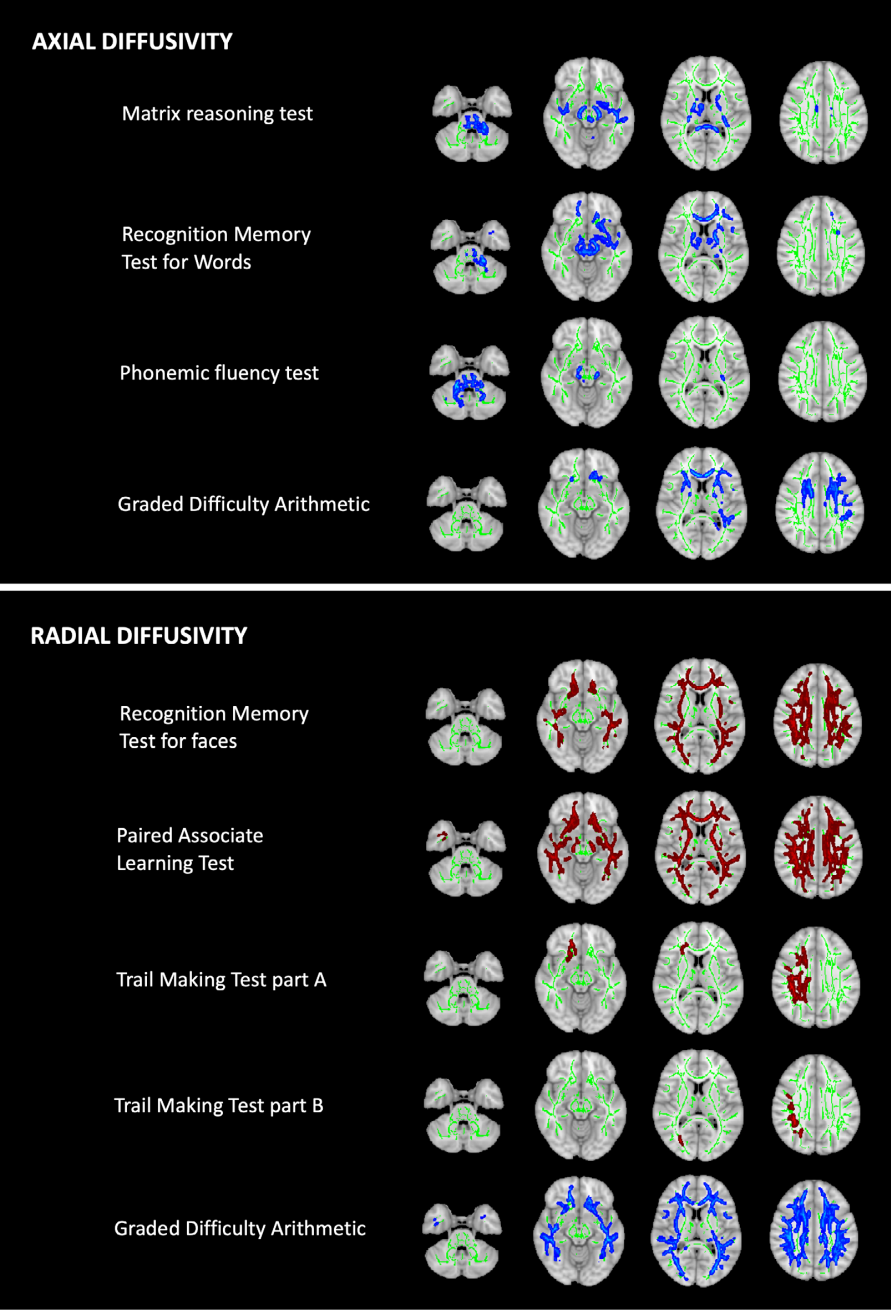
Tract‐based spatial statistics for associations with neuropsychological test scores. Tissue maps show correlations between neuropsychological test scores and axial/radial diffusivity in white matter tracts for FWE (family‐wise error)‐corrected *P‐*values <0.05. Tracts where diffusivity increases (red) or decreases (blue) with worsening cognitive performance are overlaid onto the white matter skeleton (green). Axial slices at *z* = −34, −12, 10, and 32 are shown. [Color figure can be viewed at wileyonlinelibrary.com]

Scores for several neuropsychological tests were associated with DTI parameters in WM tracts. Associations with AD and RD are shown in Figure 3, and those with FA and MD are shown in Figure S2. Poor performance on the MRT, RMTW, and FAS was associated with decreases in AD in subcortical WM tracts. Poor performance on the RMTF and PALT was associated with widespread increases in RD, with associated increases in MD and decreases in FA. Poor performance on the TMTA and TMTB was associated with increases in RD in the right corona radiata only. Unlike other neuropsychological tests, poor performance for GDA was associated with widespread *decreases* in RD, *decreases* in MD, and *increases* in FA. The aforementioned associations with scores for the RMTF, PALT, FAS, and GDA persisted after controlling for neurological severity, as shown in Figure S3.

There were no associations between neuropsychological test scores and the log_e_‐transformed volume of hyperintense signal abnormalities or susceptibility maps.

## Discussion

This is the largest cohort of patients with WD to undergo detailed neuropsychological testing, with one exception,^3^ and the only study to perform multimodal quantitative neuroimaging analyses across a range of tests. We have confirmed previous observations that some cognitive deficits are common in patients with hepatic presentations and identified neuroimaging correlates for deficits in abstract reasoning, memory, processing speed, executive function, calculation, and social cognition. In combination, our clinical and neuroradiological findings have implications for our understanding of the progression of brain pathology and phenotypic classification of WD in addition to the neuroanatomical basis for cognitive symptoms.

Group differences in our cohort confirm previous observations that patients with neurological presentations have deficits in abstract reasoning, processing speed, and visuospatial function compared to patients with hepatic presentations.^3^, ^6^, ^7^, ^8^, ^10^, ^11^ We have demonstrated for the first time that deficits in memory for faces are common in WD, and UWDRS‐N scores correlate with measures of abstract reasoning, executive function, processing speed, memory for faces, and calculation. The lack of association between neurological severity and associative learning is noteworthy and suggests that some cognitive deficits occur independently of movement disorders in WD.

In the absence of healthy controls, the frequency of poor performance relative to normative data is a useful indicator of cognitive deficits in patients with hepatic presentations. We found that poor performance on at least one test was common in patients with hepatic presentations. Scores for TMTB, a measure of cognitive flexibility, and RMTF were particularly interesting, with 24% of patients with hepatic presentations scoring more than two SDs below the mean. Seniów et al found no differences in cognitive function between neurologically asymptomatic patients, who might be considered to have a hepatic phenotype, and healthy controls.^3^ However, they did not include the RMTF, TMTB, or PALT, and analyses excluded patients with neuroimaging abnormalities.

Our clinical findings support the idea that subtle deficits in memory for faces, cognitive flexibility, and associative learning represent a distinct neurological phenotype that can emerge before the development of movement disorders and deficits in abstract reasoning and other aspects of executive function. A minority of patients with neurological presentations had preserved memory for faces, cognitive flexibility, and associative learning. This suggests that deficits in these domains are not a prerequisite for developing a movement disorder and/or may improve with treatment in some patients.

Deficits in abstract reasoning and executive function, specifically phonemic fluency, were strongly associated with lower basal ganglia volumes, particularly in the putamen. These deficits were also associated with decreasing AD, suggestive of axonal loss, in subcortical WM tracts. Task‐based functional magnetic resonance imaging (fMRI) in healthy controls shows that performing the MRT activates areas, including the dorsolateral prefrontal cortex (PFC), anterior cingulate cortex, fusiform gyri, and inferior and middle occipital cortices.^32^ Performing the FAS test is associated with activation in the left PFC, basal ganglia, and thalamus.^33^ We suspect our observations on these tests therefore reflect the disruption of frontostriatal networks that originate on the PFC and project to the caudate, putamen, and then pallidum before returning to the PFC via the thalamus.^34^

In contrast, deficits in recognition memory for faces correlated with decreased GM volumes in diffuse anterior cortical regions, including the PFC and cingulate cortices. They were also associated with diffusion abnormalities, characterized by increasing RD, in widespread WM tracts. Using [H_2_^15^O]‐PET, Kim *et al* demonstrated that performing the RMTF activates the cortical regions within the ventral stream for object recognition and dorsal stream for object lateralization in healthy individuals.^35^ Cohen *et al* recently showed that lesions causing prosopagnosia localize to a functionally connected brain network between the right fusiform gyrus and left PFC.^36^ In the absence of posterior cortical involvement, we suspect that deficits in recognition memory for faces in WD are caused by damage to the left PFC or connections between the left PFC and right fusiform gyrus.

Deficits in facial emotion recognition were associated with a similar pattern of abnormalities with decreased GM volumes in the anterior cortical and subcortical regions. In an fMRI study, facial emotion recognition was associated with the activation of brain regions, including the PFC, basal ganglia, thalamus, and amygdala.^37^ Killgore and Yurgelun‐Todd have also highlighted the importance of the anterior cingulate cortex in detecting and discriminating affective information.^38^ We suspect that damage to the orbitofrontal, insula, and/or anterior cingulate cortices accounts for deficits in social cognition in WD.

Impaired cognitive flexibility was associated with decreased volume in the occipital fusiform gyrus and right intracalcarine cortex, reflecting visual aspects of the TMTB. The same areas are activated when healthy controls perform this test.^39^ We also identified associations with basal ganglia volumes that did not persist when controlling for neurological severity. We suspect that these associations and imaging correlates for the TMTA reflect handwriting ability given the rate of information processing, independent of motor speed, has previously been shown to be preserved in WD.^40^

The contrasting associations between some neuropsychological test scores and the diffusion abnormalities we identified are interesting. Deficits in associative learning and recognition memory for faces were associated with widespread increases in RD, whereas deficits in calculation were associated with widespread decreases in RD. The diffuse nature of these abnormalities precludes any localization of these cognitive deficits beyond WM, as opposed to GM. However, the opposing nature of these associations suggests that they are driven by different pathological processes, which might include atp7b dysfunction and copper toxicity, simultaneously present throughout WM.

Drawing our findings together, distinct patterns of neuroradiological abnormalities correlate with the cognitive deficits observed in patients with hepatic presentations and those more closely associated with movement disorders. Deficits in memory for faces, cognitive flexibility, and associative learning were associated with cortical atrophy and/or increases in RD throughout the WM. Deficits in abstract reasoning and phonemic fluency were associated with atrophy localized to the basal ganglia, specifically the putamen, and decreasing AD in subcortical WM tracts, after controlling for neurological severity. These observations challenge the prevailing view that cognitive impairment in WD primarily relates to basal ganglia dysfunction and that disease progression results from more geographically widespread neuropathology, as in other neurodegenerative diseases. Instead, these data suggest that a neurological phenotype characterized by subtle cognitive deficits results from diffuse changes in cortical GM and WM, whereas a more overt neurological phenotype characterized by movement disorders and executive dysfunction emerges with worsening basal ganglia disease.

We have previously demonstrated that increasing serum non‐caeruloplasmin‐bound copper levels are associated with WM microstructural abnormalities and cortical, but not basal ganglia, atrophy in the same cohort of chronically treated patients.^17^ It has also been shown that patients with WD, including those with hepatic presentations, have ubiquitous increases in brain copper content.^41^ It is therefore plausible that our novel neurological phenotype is driven by widespread, but mostly subclinical, copper toxicity in the brain. Dysfunction of atp7b, which is expressed in neurons and glia, might also contribute.^42^ The classical neurological phenotype likely relates to a selective neuronal vulnerability within the basal ganglia present only in a subset of patients. We have previously suggested that a tendency to mishandle brain iron in response to copper toxicity might account for this.^17^ This overarching theory on the evolution of neurological involvement in WD is consistent with previous observations that subclinical psychiatric features and neuroimaging abnormalities are common in patients with hepatic presentations.^43^, ^44^ It also explains why copper indices do not differ between patients with neurological and hepatic presentations or correlate with the severity of movement disorders.^45^

Our findings also have implications for the current binary phenotypic classification of WD.^19^ Patients are divided into neurological and hepatic presentation depending on the presence or absence of overt neurological and/or psychiatric symptoms at diagnosis. Our data suggest that more comprehensive cognitive assessment at diagnosis might lead some patients with hepatic presentations to be reclassified as having a neurological presentation. The existing classification might lead some clinicians to underappreciate subtle but potentially disabling cognitive symptoms in patients with seemingly hepatic presentations. Some children and adolescents may require additional educational support, and some adults might benefit from vocational rehabilitation. We suspect that it would be more appropriate to acknowledge that all patients with WD are at risk of developing movement disorders, cognitive deficits, and psychiatric comorbidity and screen for these using appropriate rating scales or other clinical measures.

The lack of healthy controls is a major limitation in this study. We rely on previously published normative data to determine the significance of some findings and recognize that these may have been acquired under different circumstances. We cannot exclude the possibility that some neuroanatomical correlates, such as the relationship between Ekman scores and anterior cortical volumes, also exist in healthy controls, but this seems unlikely based on previous reports.^46^, ^47^ We also tested the associations between cognitive deficits and neuroimaging abnormalities only in chronically treated stable patients; we are extrapolating our findings to make inferences about the evolution of neurological involvement earlier in the disease course but acknowledge some cognitive deficits may improve or resolve with treatment.

To conclude, we have identified neuroanatomical correlates for a range of cognitive deficits observed in WD and proposed a novel theory for the evolution of neurological involvement that raises questions about the use of a binary disease classification. Confirming that newly diagnosed patients without movement disorders have deficits in cognitive flexibility, recognition memory for faces, and associative learning relative to healthy controls and that associations with cortical atrophy and WM diffusion abnormalities persist in this group would provide further supporting evidence. Our hope is that this can be tested in parallel with other imaging biomarkers for neurological involvement in clinical trials for novel treatments in the coming years.

## Full financial disclosures for the previous 12 months

S.S. has received grants from the Guarantors of Brain via the Association of British Neurologists and Wilson's Disease Support Group UK. T.T.W has received grants from the the Reta Lila Weston Medical Trust, Rostrees Trust.

## Author Roles

Design: S.S., R.C., D.L.T., G.T.G., E.A.T., O.B., J.D.R., and T.T.W.

Execution: S.S., M.Bu., M.Bo., C.H.S., G.T.G., and E.A.T.

Analysis: S.S., M.Bo., C.H.S., J.A.‐C., J.D.R., and T.T.W.

Writing: S.S., M.Bo., D.L.T., G.T.G., O.B., J.D.R., and T.T.W.

Editing: J.D.R. and T.T.W.

## Supporting information


**APPENDIX S1.** Supporting Information

## Data Availability

Anonymised data are available on request to the corresponding author.
