# Supplementary material for: Neuroimaging Correlates of Cognitive Deficits in Wilson's Disease
Source: Mov Disord. 2022 Jun 20;37(8):1728–38. doi: 10.1002/mds.29123 (PMC9542291; doi:10.1002/mds.29123)

Supplementary table 1. Neuropsychological test battery and normative sources

Domain	Test	Normative data source
Abstract reasoning	Weschler Abbreviated Scale of Intelligence matrix reasoning test	Weschler Abbreviated Scale of Intelligence | Second edition. *Pearson* 2011.
Language	National Adult Reading Test	Warrington. The Graded Naming test: A restandardisation. *Neuropsychological Rehabilitation* 1997;7(2):143-6.
	Graded Naming Test	
Memory	Recognition Memory Test for Faces	Warrington. The Camden Memory Tests Manual. *Psychological Press* 1996.
	Recognition Memory Test for Words	
	Paired Associate Learning test	
Processing speed	Trail Making Test part A	Tombaugh. Trail Making Test A and B: Normative data stratified by age and education. *Arch Clin Neuropsychol* 2004;19(2):203-14.
Executive function	Weschler Memory Scale Revised Digit Span Backwards	Genetic Frontotemporal Dementia Initiative cohort (unpublished).
	Phonemic fluency test	Tombaugh. Normative data stratified by age and education for two measures of verbal fluency: FAS and animal naming. *Arch Clin Neuropsychol* 1999;14(2):167-77.
	Semantic fluency test	
	Delis-Kaplan Executive Function System Color-Word Interference Test	Delis-Kaplan Executive Function System. *Pearson* 2001.
	Trail Making Test part B	Tombaugh. Trail Making Test A and B: Normative data stratified by age and education. *Arch Clin Neuropsychol* 2004;19(2):203-14.
	Weschler Adult Intelligence Scale Digit Symbol test	Weschler Abbreviated Scale of Intelligence | Second edition. *Pearson* 2011.
Calculation	Graded Difficulty Arithmetic Test	Jackson and Warrington. Arithmetic skills in patients with unilateral cerebral lesions. *Cortex* 1986l22(4):611-20.
Visuoperceptual	Visual Object and Space Perception battery Fragmented Letters test	Visual Object and Space Perception battery. *Pearson* 1991.
Visuospatial	Visual Object and Space Perception battery Number Location test	
Social	Ekman Facial Emotion Recognition test	Genetic Frontotemporal Dementia Initiative cohort (unpublished).

Supplementary table 2. Pulse sequence parameters

	**3D T1**	**3D FLAIR**	**DWI**	**3D SWI**	**B0 fieldmap**
Pulse sequence	MPRAGE	IR-SPACE	Double refocused PGSE-EPI	3D gradient echo	2D gradient echo
Voxel resolution (mm^3)	1.1 x 1.1 x 1.1	1.0 x 1.0 x 1.0	2.5 x 2.5 x 2.5	1.0 x 1.0 x 1.0	3.0 x 3.0 x 3.0
Matrix size	256 x 256 x 208	256 x 256 x 192	96 x 96 x 59	256 x 192 x 176	64 x 64 x 55
Field of view (mm)	282 x 282 x 229	256 x 256 x 192	240 x 240 x 148	256 x 192 x 176	192 x 192 x 165
Orientation	Sagittal	Sagittal	Axial	Axial	Axial
Phase-encoding direction	A >> P	A >> P	A >> P	R >> L	R >> L
Echo time, TE (ms)	2.93	403	90	4.94/9.88/14.82/19.76/24.70	4.92/7.38
Recovery time, TR (ms)	2000	4800	7300	30	688
Flip angle (degrees)	8	Variable	-	15	60
Acquisition bandwidth (Hz/Px)	240	751	1578	280/260/260/260/260	260
Parallel imaging (GRAPPA acceleration factor)	2	3	2	3	None
Total scan time	5 min 6 sec	4 min 54 sec	8 min 47 sec	4 min 9 sec	1 min 31 sec
Other sequence specific parameters	Inversion time, IR, 850 ms	Inversion time, TI, 1650 ms SPACE turbo factor 243	Twice-refocused 2D multi-slice SE-EPI readout, b = 1000 s/mm^2 for diffusion encoding along 64 orientations. Five interspersed b = 0 s/mm^3 scans.	Partial Fourier 6/8 Monopolar readout Flow compensation for first echo.	2D multi-slice

GRAPPA, generalised autocalibrating partial parallel acquisition; IR-SPACE, inversion recovery – sampling perfection with application optimised contrast using different flip angle evolutions; MPRAGE, magnetisation prepared rapid gradient echo; PGSE-EPI, pulsed-gradient spin echo – echo planar imaging

Imaging acquisition, processing and analysis

T1-weighted (structural) imaging

Voxel-based morphometry (VBM) was performed using Statistical Parametric Mapping (SPM12, version 7771, http://www.fil.ion.ucl.ac.uk/spm) to identify clusters where decreasing grey matter (GM) volume were associated where decreasing cognitive performance.[33] T1-weighted (structural) images were segmented into GM, WM and cerebrospinal fluid (CSF) using standard procedures and spatially normalised using the fast-diffeomorphic image registration algorithm.[34] GM and WM segments were transformed into MNI152 space (Montreal Neurological Institute, McGill University, Canada), modulated and smoothed using a Gaussian kernel with 8 mm full-width at half maximum to create pre-processed GM tissue maps. All segmentations were visually checked for quality. The pre-processed tissue maps were fitted to multiple regression analyses to identify associations with neuropsychological test scores. Total intracranial volume (TIV), calculated in SPM, was included as a nuisance covariate, in addition to age and sex.[35] Statistical thresholds were set at $P < 0.05$ for family-wise-error (FWE) correction and then lowered to uncorrected $P < 0.001$. A minimum cluster size of 20 voxels was set and thresholded statistical maps were overlaid onto the study-wise mean template.

We conducted a separate region-of-interest (ROI) analysis to assess atrophy in specific subcortical structures. T1-weighted images were bias-corrected and parcellated using the geodesic information flow (GIF) pipeline,[36] based on atlas propagation and label fusion. The brainstem was subsequently segmented using a customized version of a FreeSurfer module.[37] We did not manually correct the automatic segmentation of any ROI. The volume of eight subcortical ROI including the caudate, putamen, pallidum, thalamus, amygdala, midbrain, pons and cerebellum, were extracted and expressed as a percentage of total intracranial volume (TIV), calculated in SPM. All segmentations were visually checked for quality. Linear regression was used to identify associations with neuropsychological test scores. P values for coefficients of interest both with and without false discovery rate (FDR) correction were calculated in R (version 3.6.0, http://www.R-project.org).

FLAIR imaging

WMHs were segmented using Bayesian model selection, an automated lesion segmentation tool applied to rigidly co-registered T1-weighted (structural) and FLAIR sequences.[38] A

Gaussian mixture model with dynamically evolving number of components was fit to the data, modelling simultaneously healthy and non-expected observations. WMH-related measures were introduced to the model through subject-specific statistical atlases obtained using the GIF pipeline. After convergence, the model was used to select candidate lesion voxels whose aggregation in connected components was automatically classified as lesion or artefact. WMH segmentations were then visually inspected and flagged if there were significant segmentation errors. This quality control stage was used to make improvements to the automated WMH segmentation, thereby maximising the number of usable segmentations.

The volume of WMHs within 40 anatomically-defined regions were calculated for each participant.[39] WM was separated into four equidistant layers between the ventricular surface and the cortical GM/WM interface. These were then divided into left and right frontal, temporal, parietal and occipital lobes using the GIF parcellation. The basal ganglia and infratentorial regions were considered separately. The volume of WMHs within each region was $\log_e$-transformed to reduce skewness. A linear regression model was used to identify associations with neuropsychological test scores. TIV was included as a covariate of no interest, in addition to age and sex. P values for coefficients of interest were calculated with FDR correction in R and these were summarised in bullseye plots to illustrate their anatomical distribution.[39]

Diffusion-weighted imaging

The Functional MRI of the Brain Software Library (FSL, version 6.0.3, https://fsl.fmrib.ox.ac.uk/fsl) was used to pre-process DWI data prior to fitting the single tensor model, resulting in volumetric diffusion tensor imaging (DTI) data. DTI datasets were then analysed using tract-based spatial statistics (TBSS).[40] Pre-processing included EDDY to correct for motion and eddy-currents with outlier replacement enabled. FUGUE was applied to correct for distortions using fieldmaps. Tensors were fitted using DTIFIT and fractional anisotropy (FA), mean diffusivity (MD), axial diffusivity (AD) and radial diffusivity (RD) maps were generated, skeletonised and aligned using TBSS. Design matrices for identifying associations with neuropsychological test scores were generated using the general linear model. Finally, RANDOMISE was used to perform nonparametric permutation analyses based on each design matrix. Covariates were mean-centred and 10,000 permutations of the data were carried out. The threshold-free cluster enhancement algorithm was used to identify clusters of voxels with a FWE corrected P value < 0.05.[41] Clusters of increased or decreased FA, MD, AD and

RD were then overlaid onto a mask of the WM skeleton (created using the mean skeletonised FA map) and the MNI152 template.

Susceptibility-weighted imaging

Quantitative susceptibility maps (QSM) were reconstructed from susceptibility-weighted images using a Multi-Scale Dipole Inversion (MSDI)-based pipeline for coil-combined, multi-gradient echo data in QSMbox (https://gitlab.com/acostaj/QSMbox).[42] Pre-processing steps included unwrapping of complex 3D phase data using a discrete Laplacian method followed by background field removal using Laplacian boundary extraction and variable spherical mean filtering. All steps were applied using default settings. Whole-brain analyses were performed with the QSMexplorer pipeline (https://gitlab.com/acostaj/QSMexplorer).[43] A study-wise space was created from T1-weighted sequences using Advanced Normalisation Tools (ANTs). Bias-corrected magnitude images were then used to transform the quantitative susceptibility maps to the study-wise space. Absolute susceptibility maps smoothed with a 3 mm standard deviation 3D Gaussian kernel were used to identify associations with neuropsychological test scores in stable patients. RANDOMISE was used to perform nonparametric permutation analyses based on each design matrix. Covariates were mean-centred and 10,000 permutations were performed. The GM segment generated in SPM12 was used to mask the absolute maps. Threshold-free cluster enhancement was enabled to identify clusters of voxels with a family-wise error corrected P value < 0.05. Clusters were then overlaid (for result visualisation) onto the study-wise template.

Supplementary table 3. Demographic and clinical characteristics

	**All (n=40)**	**Hepatic (n=17)**	**Neurological (n=23)**	**P value**	**Stable (n=35)**	**Active (n=5)**	**P value**
Age, mean (SD), yr	43 (14)	42 (15)	44 (14)	0.77	44 (14)	39 (17)	0.47
Sex, n (%)							
Female	20 (50%)	8 (47%)	12 (52%)	0.75	19 (54%)	1 (20%)	0.15
Male	20 (50%)	9 (53%)	11 (48%)		16 (46%)	4 (80%)	
Years of education, mean (SD)	15 (3)	15 (3)	14 (3)	0.35	15 (3)	14 (3)	0.49
Ethnicity, n							
White	28	12	16	0.07	25	3	0.84
Asian/Asian British	7	1	6		6	1	
Other ethnic group [a]	5	4	1		4	1	
ATP7B genotype							
Homozygous mutations	9	4	5		8	1	
Compound heterozygous mutations	17	10	7		14	3	
Single heterozygous mutation	1	0	1		1	0	
Not tested/results unavailable	13	3	10		12	1	
Age at symptom onset, mean (SD), yr	19 (12)	20 (17)	19 (9)	0.75	19 (13)	19 (6)	0.82
Disease duration, mean (SD), yr	23 (15)	20 (15)	25 (16)	0.37	24 (15)	20 (20)	0.56
Evidence of cirrhosis, n (%) [b]	17 (43%)	7 (41%)	10 (43%)	0.88	14 (40%)	3 (60%)	0.40
Alanine transaminase, median (IQR), IU/L	31 (17-46)	41 (27-47)	20 (17-43)	0.08	27 (17-46)	45 (32-73)	0.15
Kayser-Fleischer rings, n	16/34	3/13	13/21	0.04	13/30	3/4	0.48
Treatment, n (%)							
Penicillamine	26 (65%)	9 (53%)	17 (74%)	0.39	23 (66%)	3 (60%)	0.82
Trientine	9 (23%)	5 (29%)	4 (17%)		7 (20%)	2 (40%)	
Zinc	1 (3%)	1 (6%)	0 (0%)		1 (3%)	0 (0%)	
Combination	1 (3%)	0 (0%)	1 (4%)		1 (3%)	0 (0%)	
Liver transplantation	3 (8%)	2 (12%)	1 (4%)		3 (95)	0 (0%)	

[a] *Ethnic groups other than White and Asian/Asian British were grouped to preserve the anonymity of some participants.*
[b] *Evidence of cirrhosis was based on previous imaging or histopathological results.*

Supplementary table 4. Frequency of poor performance in neuropsychological tests

Domain	Test	All	Hepatic	Neurological	Stable	Active
Abstract reasoning	MRT	3% (1/39)	0% (0/17)	5% (1/22)	3% (1/35)	**25% (**1/4)
Language	NART	3% (1/33)	7% (1/15)	0% (0/18)	4% (1/30)	0% (0/3)
	GNT	**15%** (5/33)	**14%** (2/15)	**17%** (3/18)	**17%** (5/30)	0% (0/3)
Memory	RMTF	**36%** (14/39)	**24%** (4/17)	**45%** (10/22)	**31%** (11/35)	**75%** (3/4)
	RMTW	5% (2/39)	6% (1/17)	5% (1/21)	3% (1/35)	**25%** (1/4)
	PALT	**19%** (7/37)	**12%** (2/17)	**25%** (5/20)	**15%** (5/34)	**67%** (2/3)
Processing speed	TMTA	**24%** (9/38)	**12%** (2/17)	**33%** (7/21)	**18%** (6/34)	**75%** (3/4)
Executive function	DSB	3% (1/38)	0% (0/17)	5% (1/21)	0% (0/34)	**25%** (1/4)
	FAS	5% (2/38)	0% (0/17)	9% (2/21)	0% (0/34)	**50%** (2/4)
	Animals	0% (0/38)	0% (0/17)	0% (0/21)	0% (0/34)	0% (0/4)
	DKEFSI	8% (3/37)	6% (1/17)	**10%** (2/20)	6% (2/34)	**33%** (1/3)
	TMTB	**34%** (13/38)	**24%** (4/17)	**43%** (9/21)	**29%** (10/34)	**75%** (3/4)
	DSym	5% (2/38)	0% (0/17)	9% (2/21)	0% (0/34)	**50%** (2/4)
Calculation	GDA	3% (1/38)	0% (0/17)	5% (1/21)	0% (0/34)	**25%** (1/4)
Visuoperceptual	VOSPFL	0% (0/39)	0% (0/17)	0% (0/22)	0% (0/35)	0% (0/4)
Visuospatial	VOSPNL	8% (3/39)	0% (0/17)	**14%** (3/22)	6% (2/35)	**25%** (1/4)
Social	Ekman	8% (3/39)	0% (0/17)	**14%** (3/22)	6% (2/35)	**25%** (1/4)

Percentages of participants who scored more than two standard deviations below the mean are shown for each neuropsychological test. Percentages above 10% are highlighted in bold.

Animals, semantic fluency test; PALT, Paired Associate Learning test; DKEFSI, Delis-Kaplan Execution Function System Color-Word Interference subtest; DSB, Weschler Memory Scale Revised Digit Span Backwards; DSym, Weschler Adult Intelligence Scale Digit Symbol test; Ekman, Ekman Facial Emotion Recognition test; FAS, phonemic fluency test; GDA, Graded Difficulty Arithmetic test; GNT, Graded Naming Test; MRT, Weschler Abbreviated Scale of Intelligence matrix reasoning test; NART, National Adult Reasoning Test; RMTF, Recognition Memory Test for Faces; RMTW, Recognition Memory Test for Words; TMTA, Trail Making Test part A; TMTB, Trail Making Test part B; UWDRS-N, Unified Wilson's Disease Rating Scale neurological examination subscore; VOSPFL, Visual Object and Space Perception battery Fragmented Letters test; VOSPNL, Visual Object and Space Perception battery Number Location test.

Supplementary table 5. Associations between ROI volumes and neuropsychological test scores with UWDRS-N as covariate

Domain	Test	Caudate	Putamen	Pallidum	Thalamus	Amygdala	Midbrain	Pons	Cerebellum
Abstract reasoning	MRT	0.08	**0.005****	0.03*	0.15	0.51	0.43	0.44	0.03*
Language	NART	0.16	0.17	0.12	0.52	0.70	0.53	0.76	0.28
	GNT	0.05*	0.07	0.02*	0.06	0.90	0.28	0.32	0.10
Memory	RMTF	0.27	0.24	0.42	0.90	0.73	0.47	0.47	0.35
	RMTW	0.75	0.28	0.29	0.39	0.80	0.35	0.57	0.31
	PALT	0.06	0.15	0.31	0.94	0.51	0.77	0.47	0.57
Processing speed	TMTA	0.14	0.37	0.40	0.64	0.97	0.80	0.59	0.92
Executive function	DSB	0.07	0.02*	0.02*	0.30	0.67	0.64	0.51	0.34
	FAS	0.02*	**0.004****	0.02*	0.12	0.24	0.03*	0.03*	0.07
	Animals	0.21	0.43	0.35	0.97	0.77	0.62	0.75	0.06
	DKEFSI	0.25	0.13	0.24	0.58	0.41	0.59	0.66	0.27
	TMTB	0.01*	0.01*	0.007**	0.01*	0.90	0.005**	0.01*	0.74
	DSym	0.07	0.09	0.09	0.21	0.59	0.16	0.17	0.20
Calculation	GDA	0.19	0.25	0.24	0.89	0.89	0.81	0.98	0.50
Visuoperceptual	VOSPFL	0.41	0.51	0.60	0.38	0.37	0.48	0.59	0.30
Visuospatial	VOSPNL	0.69	0.65	0.88	0.40	0.65	0.53	0.61	0.36
Social	Ekman	0.69	0.49	0.95	0.95	0.38	0.33	0.77	0.56

*P values for coefficients when testing associations between neuropsychological test scores and ROI volumes using linear regression with UWDRS-N as a covariate are shown. Corresponding coefficients where P < 0.05 were positive. * = P value <0.05; ** = P value <0.01; *** = P value <0.001. P values less than 0.05 after FDR correction are highlighted in bold.*

Animals, semantic fluency test; PALT, Paired Associate Learning test; DKEFSI, Delis-Kaplan Execution Function System Color-Word Interference subtest; DSB, Weschler Memory Scale Revised Digit Span Backwards; DSym, Weschler Adult Intelligence Scale Digit Symbol test; Ekman, Ekman Facial Emotion Recognition test; FAS, phonemic fluency test; FDR, false discovery rate; GDA, Graded Difficulty Arithmetic test; GNT, Graded Naming Test; MRT, Weschler Abbreviated Scale of Intelligence matrix reasoning test; NART, National Adult Reasoning Test; RMTF, Recognition Memory Test for Faces; RMTW, Recognition Memory Test for Words; ROI, region of interest; TMTA, Trail Making Test part A; TMTB, Trail Making Test part B; UWDRS-N, Unified Wilson's Disease Rating Scale neurological examination subscore; VOSPFL, Visual Object and Space Perception battery Fragmented Letters test; VOSPNL, Visual Object and Space Perception battery Number Location test.

Supplementary figure 1. Voxel-based morphometry for associations with neuropsychological test scores with UWDRS-N subscores as a covariate. Tissue maps show clusters where grey matter volumes decrease with worsening cognitive performance for FWE-corrected P values < 0.05. Clusters are overlaid onto the study-wise mean template. For visualisation purposes one slice in each of the sagittal (x), coronal (y) and axial (z) planes was selected and MNI coordinates are provided.

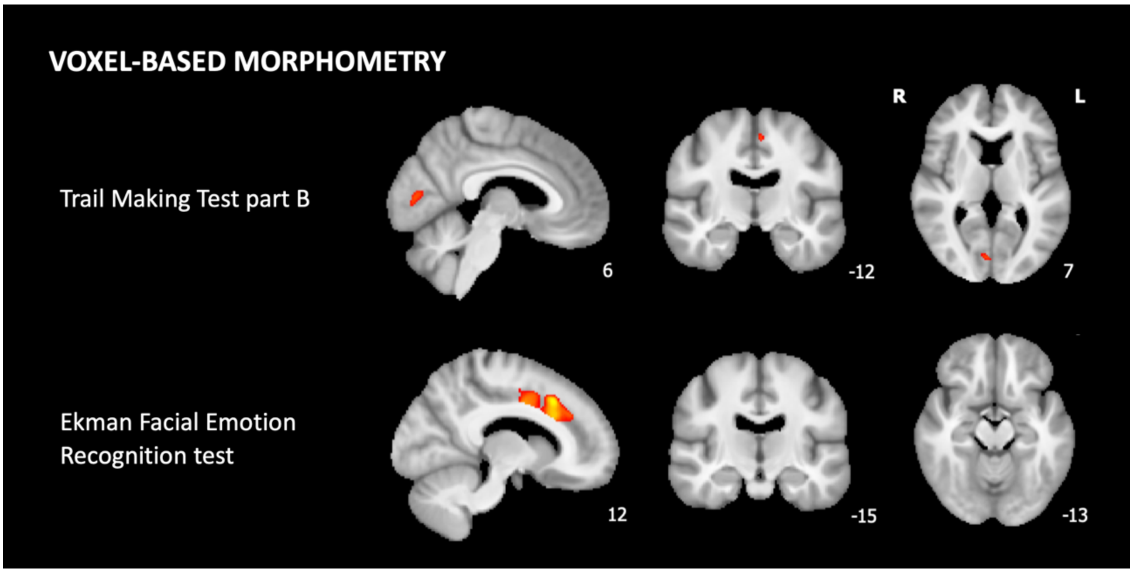

Supplementary table 6. Voxel-based morphometry statistics

**Analysis**		**Size**	**TFCE**	***P* FWE**	***P* uncorr**	**x**	**y**	**z**
MRT	R putamen, insula and orbitofrontal cortices	2073	2031	0.012	0.001	30	4	-12
	L cerebellum	23	1444	0.047	0.001	-38	-44	-44
RMTF	Bilateral cingulate, paracingulate and insula cortices, middle frontal gyri, supplementary motor areas, caudate and putamen	22190	2202	0.008	<0.001	9	-6	56
	R superior and middle temporal gyri and subcallosal and opercular cortices	1170	1598	0.033	0.001	56	-32	-2
	R cerebellum	374	1499	0.041	0.001	9	-56	-8
	Dorsal midbrain	585	1499	0.041	0.001	0	-39	-18
	L middle frontal gyrus	215	1479	0.043	<0.001	-24	32	36
	R frontal pole	141	1469	0.044	<0.001	21	57	4
	R insula cortex	179	1464	0.045	0.002	44	-8	3
	R insula cortex	24	1427	0.049	0.001	32	-18	6
Animals	L cerebellum	601	1806	0.033	0.001	-21	-56	-60
TMTB	L insula cortex	333	1550	0.042	0.001	-33	-22	9
	L supplementary motor area	140	1527	0.044	0.001	-9	-18	60
	L precuneus	59	1500	0.047	0.001	-3	-66	21
	R intracalcarine cortex	190	1499	0.047	0.001	4	-78	10
TMTB with UWDRS-N	L occipital fusiform gyrus	76	1523	0.044	0.001	-22	-88	-15
	R intracalcarine cortex	84	1482	0.048	0.001	6	-80	9
	L supplementary motor area	34	1482	0.048	0.001	-3	-9	54
Ekman	Bilateral cingulate and paracingulate cortices and supplementary motor areas and L superior frontal gyrus	5493	2008	0.012	<0.001	9	22	39
	Bilateral cerebellum	8096	1878	0.016	<0.001	2	-74	-36
	R central opercular and insula cortices and putamen, L temporal fusiform cortex and bilateral orbitofrontal cortices and hippocampi	9604	1807	0.019	<0.001	62	8	16
	L pre- and post-central gyri	734	1655	0.027	<0.001	-52	-15	45
	R middle frontal gyrus and frontal pole	600	1528	0.037	<0.001	32	27	38
	L cerebellum	763	1489	0.041	0.002	-34	-75	-34
	R pre- and post-central gyri	329	1470	0.043	<0.001	52	-16	44
	R middle frontal gyrus	200	1457	0.044	<0.001	50	9	42
	R middle frontal and pre-central gyri	103	1450	0.045	0.001	32	-2	56
	L pre-central gyrus	36	1440	0.046	<0.001	-51	8	27
	L insula cortex	73	1427	0.048	0.001	-42	-6	2
	L post-central gyrus	28	1410	0.050	<0.001	-63	-8	24
Ekman with UWDRS-N	Bilateral cingulate and paracingulate cortices	1961	1673	0.025	<0.001	9	22	39
	R central opercular cortex	168	1452	0.044	<0.001	60	8	16

Animals, semantic fluency test; Ekman, Ekman Facial Emotion Recognition test; MRT, Weschler Abbreviated Scale of Intelligence matrix reasoning test; RMTF, Recognition Memory Test for Faces; TFCE, threshold-free cluster enhancement; TMTB, Trail Making Test part B; UWDRS-N, Unified Wilson's Disease Rating Scale neurological examination subscore.

Supplementary figure 2. Tract-based spatial statistics for associations with neuropsychological test scores. Tissue maps show correlations between neuropsychological tests scores and mean diffusivity/fractional anisotropy in white matter tracts for FWE-corrected P values < 0.05. Tracts where diffusion parameters increase (red) or decrease (blue) with worsening cognitive performance are overlaid onto the white matter skeleton (green). Axial slices at z = -34, -12, 10 and 32 are shown.

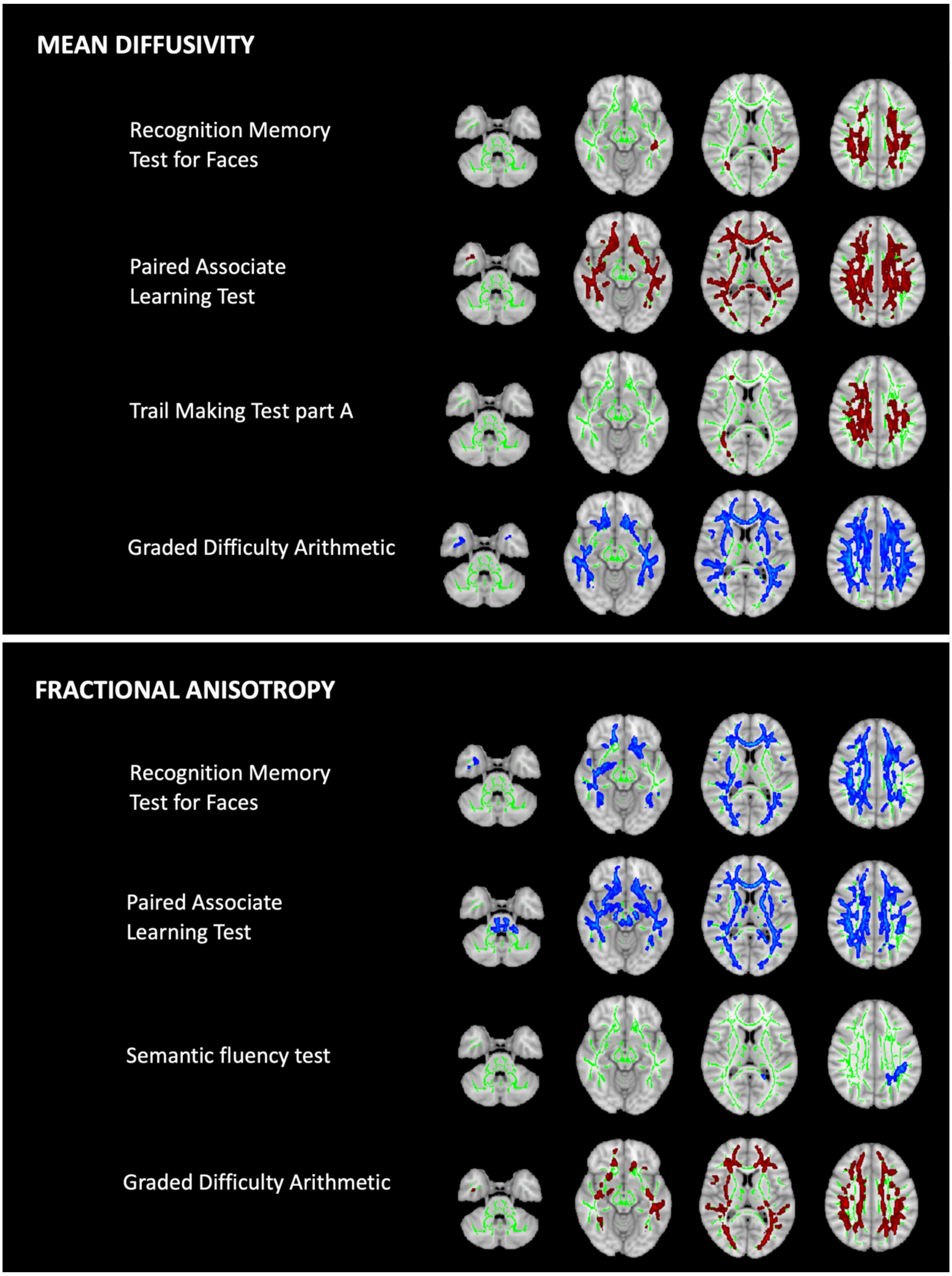

Supplementary figure 3. Tract-based spatial statistics for associations with neuropsychological test scores, as described in figure 3 and supplementary figure 2, after including UWDRS-N subscores as a covariate.

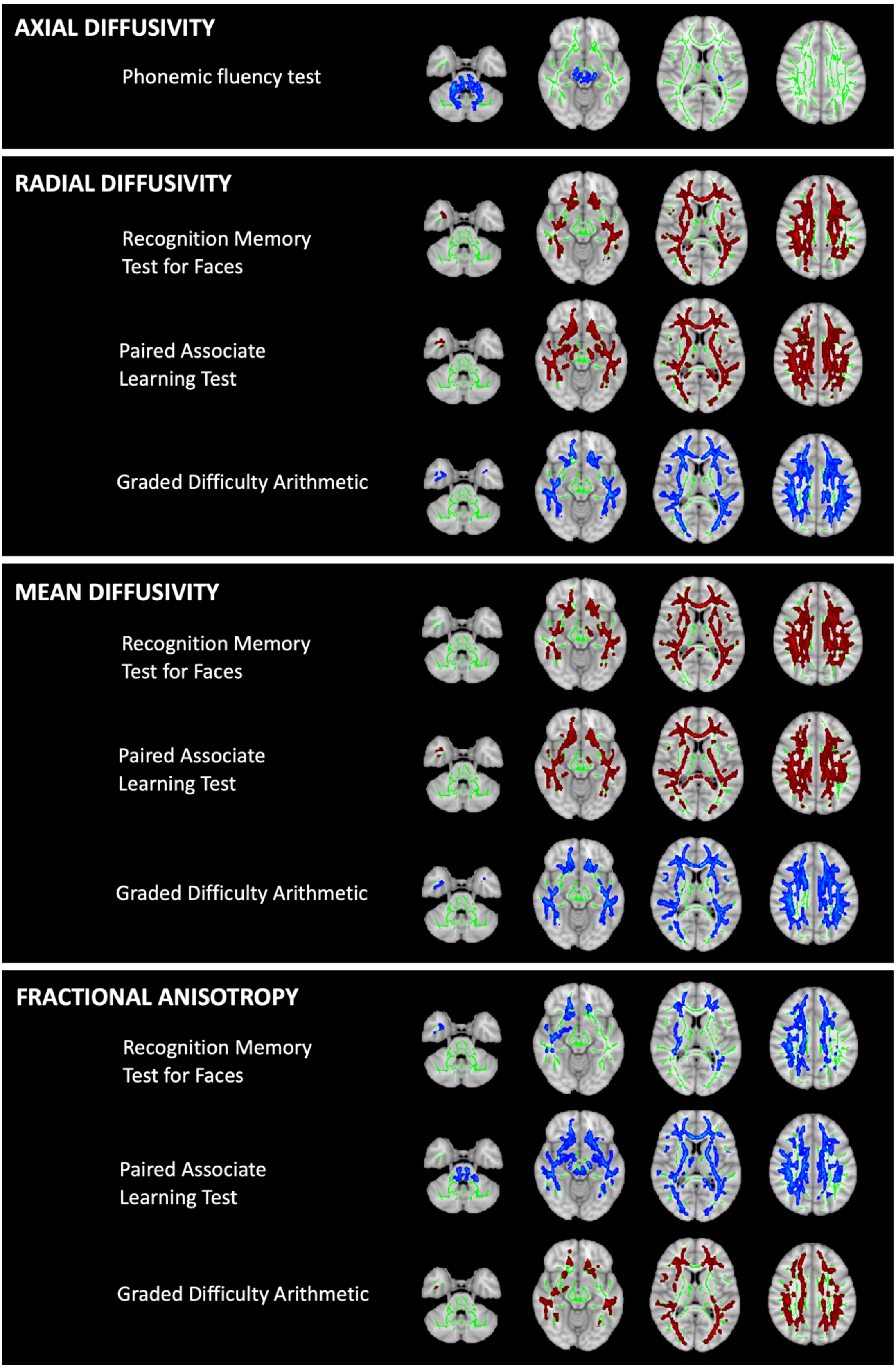

Supplement: Supplementary file 1 — APPENDIX S1. Supporting Information [file MDS-37-1728-s001.pdf]
